# Pregnancy and Maternal Behavior Induce Changes in Glia, Glutamate and Its Metabolism within the Cingulate Cortex

**DOI:** 10.1371/journal.pone.0023529

**Published:** 2011-09-06

**Authors:** Natalina Salmaso, Marie-Pierre Cossette, Barbara Woodside

**Affiliations:** 1 Center for Studies in Behavioral Neurobiology, Concordia University, Montreal, Quebec, Canada; 2 Yale School of Medicine, Yale University, New Haven, Connecticut, United States of America; Institut National de la Santé et de la Recherche Médicale, France

## Abstract

An upregulation of the astrocytic proteins GFAP and bFGF within area 2 of the cingulate cortex (Cg2) occurs within 3 hours of parturition in rats. These changes are the result of an interaction between hormonal state and maternal experience and are associated with increased dendritic spine density in this area. Here, we examined whether this upregulation of astrocytic proteins generalized to other glial markers and, in particular those associated with glutamate metabolism. We chose glial markers commonly used to reflect different aspects of glial function: vimentin, like GFAP, is a marker of intermediate filaments; glutamine synthetase (GS), and S-100beta, are used as markers for mature astrocytes and GS has also been used as a specific marker for glutamatergic enzymatic activity. In addition, we examined levels of proteins associated with glutamine synthetase, glutamate, glutamine and two excitatory amino acid transporters found in astrocytes, glt-1 and glast. S100beta immunoreactivity did not vary with reproductive state in either Cg2 or MPOA suggesting no change in the number of mature astrocytes across these conditions. Vimentin-ir did not differ across groups in Cg2, but expression of this protein decreased from Day 1 postpartum onwards in the MPOA. By contrast, GS-ir was increased within 24 h postpartum in Cg2 but not MPOA and similarly to GFAP and bFGF this upregulation of GS resulted from an interaction between hormonal state and maternal experience. Within Cg2, upregulation of GS was not accompanied by changes in the astrocytic glutamatergic transporters, glt-1 and glast, however, an increase in both glutamate and glutamine proteins were observed within the Cg2 of postpartum animals. Together, these changes suggest postpartum upregulation of glutamatergic activity and metabolism within Cg2 that is stimulated by pregnancy hormones and maternal experience.

## Introduction

There is extensive remodeling of neural circuits in postpartum mammals. In rats, these include changes in dopamine, oxytocin, vasopressin and corticotropin releasing hormone neurotransmitter systems [1], [2], [3], [4]. Changes associated with the postpartum period are not limited to neurons, however; astrocytic changes have also been documented in several hypothalamic areas [5]. Theodosis and her colleagues, for example, have documented morphological changes in astrocytes within the supraoptic (SON) and paraventricular (PVN) nuclei of the hypothalamus that have been associated with the bolus release of oxytocin that occurs during milk letdown [6], [7], [8], [9]. Astrocytic changes have also been shown in the medial preoptic area (MPOA), a structure necessary for normal expression of maternal behavior. Featherstone et al (2000) have shown higher numbers of astrocytes within the MPOA of multiparous rats recently exposed to pups than in non-pup exposed multiparous females [10].

There are few documented changes in astrocytes associated with reproductive state outside of the hypothalamic area. However, we have recently shown changes in astrocytic protein expression in the cingulate cortex area 2 (Cg2) of postpartum females [11], [12], [13]. There is a robust increase in the number of astrocytes that express glial fibrillary acidic protein (GFAP) and basic fibroblast growth factor (bFGF) in the Cg2 of lactating females compared to cycling females. This upregulation begins late in pregnancy, remains elevated throughout lactation, and for at least 8 days following pup removal, suggesting long-lasting plastic changes in glia within this region [11], [12], [13]. The dramatic increase in GFAP that we observed in Cg2 was specific to that area and was not seen either in adjacent areas of the cortex, such as Cg1, or in the MPOA [11]. Furthermore, although there are small but significant fluctuations in bFGF and GFAP levels within Cg2 across the estrous cycle, even the highest levels observed remained significantly below those levels seen postpartum [13].

GFAP is one of the most commonly used markers for astrocytes and, not surprisingly, most studies of the effects of reproductive state on morphological remodeling of astrocytes, including our own, have used GFAP as an immunocytochemical marker [11], [13], [14], [15], [16], [17]. GFAP is an intermediate filament protein and is typically upregulated in reactive astrocytes. It is clear, however, that the use of this single marker does not necessarily provide a complete picture of the modifications that may be occurring within astrocytic populations. For example, we have demonstrated that in contrast to lactating animals, cycling animals consistently show few GFAP-ir astrocytes in Cg2 [13]. This phenomenon could reflect fewer astrocytes in the Cg2 of cycling females, however it is more likely that the increase in bFGF and GFAP within Cg2 of lactating animals is due to changes in the levels of GFAP and bFGF proteins expressed by astrocytes. Indeed, increases in bFGF and GFAP expression are associated with reactivity in astrocytes. For example, increases in both are seen in response to injury such as ischemic stroke, and in non-pathological increases in glutamate, such as during learning [18], [19], [20], [21]. It is therefore possible that the number of astrocytes is not affected by reproductive state but that the changes in hormonal and sensory milieu of late pregnancy and lactation alter inputs into Cg2 that lead to changes in astrocytic state that are themselves reflected in the expression of GFAP.

Several other common markers for astrocytes exist, including S-100beta, glutamine synthetase (GS) and vimentin, and each of these proteins is associated with particular astrocytic states and functions. S100beta is a calcium binding protein that acts as a growth factor and potent mitogen of astrocytes [22] whereas GS is an enzyme important for the conversion of glutamate to glutamine [20]. Both of these proteins, however, are typically used as markers of mature astrocytes. Conversely, vimentin is an intermediate filament protein typically expressed in radial glia or immature astrocytes.

In the current studies, we used these three astrocytic markers to further investigate the state of astrocytes both within Cg2 and the medial preoptic area (MPOA) in cycling rats and across lactation. We included the MPOA because this area is critical for the onset of maternal behavior (Numan Woodside, 2010) and is activated by sensory stimuli from offspring (Fleming, Walsh, 1994, Lonstein, Simmons, Swan and Stern, 1998). Moreover, in earlier studies, an increase in GFAP-ir in the MPOA was observed in postpartum rats. [10], [11]. Because in the first studies reported here we observed a robust upregulation of glutamine synthetase in the Cg2 shortly after the onset of maternal behavior, we subsequently investigated whether levels of other proteins in the glutamate/glutamine cycle including glutamate and its metabolite, glutamine, and two astrocytic excitatory amino acid transporters (EAAT), glt-1 and glast varied with reproductive state.

## Methods

All procedures were approved by the Animal Research Ethics Committee of Concordia University under the guidelines of the Canada Council on Animal Care (ethics approval number: AREC-2004-WOOD and AREC-2007-WOOD).

### Animals

Female Wistar rats were obtained from Charles River Breeding Farms, St. Constant, Quebec and weighed 220–240 grams at the start of each experiment. Rats were maintained on a 12 hour light/dark cycle (lights on at 07:00/lights off at 19:00) at an ambient temperature of 20±2 degrees Celsius. All rats were given *ad libitum* access to rat chow (Agway Ltd) and water throughout the experiment.

### Cycling groups

Vaginal smears were taken daily from all rats in the cycling groups. Only rats that showed at least two consecutive 4–5 day estrous cycles were included in these studies and these were perfused on the metestrus day of their cycle.

### Lactating groups

All rats were housed in group cages (five females to one male) until mating was confirmed by the presence of sperm in the vaginal smear after which they were individually housed. Rats were perfused 3 hours (H3), 24 hours (D1), four days (D4) or sixteen days (D16) after the birth of the first pup. Rats in the Day 4 and 16 postpartum groups were housed with their litters (culled to 8 pups on Day 1 postpartum) until perfused. Although no formal measure of maternal behavior was taken, pups were healthy, had milk bands, and mothers were observed crouching over and nursing their litters.

To investigate the effects of exposure to a hormone profile mimicking that of late pregnancy with or without pup exposure and maternal experience, ovariectomized virgin rats were treated with a hormonal regime that mimics the hormonal profile of pregnancy and allowed three hours of maternal experience. The levels of glutamine synthetase in Cg2 were then compared among six groups of rats: ovariectomized rats given implants of estrogen and progesterone with subsequent progesterone removal without exposure to pups (HP), ovariectomized rats given the same hormonal priming and exposed to pups that subsequently showed full maternal behavior (HP Maternal), hormonally primed rats yoked for duration of pup exposure to the previous group but that did not show full maternal behavior (HP Not Maternal), and postpartum rats perfused three hours (H3) or 16 days after birth (D16).

### Ovariectomy

Rats in all hormonally-primed groups were anesthetized with a ketamine/xylazine mixture (5.7 mg ketamine and 0.86 mg xylazine/100 g of body weight, ip) and ovaries were removed through bilateral dorsal incisions powdered with a topical antibiotic (Cicatrin™) prior to suturing.

### Hormone Administration

Hormones were administered via silastic implants according to a regimen originally described by Bridges et al. (1984). Two mm lengths of silastic tubing (Dow Corning, .78″ ID×.125″ OD) were packed with crystalline 17*B* estradiol (Sigma- Aldrich, Canada) and 30 mm lengths of silastic tubing were filled with crystalline progesterone (Sigma- Aldrich, Canada). Both ends of each implant were sealed with medical adhesive silicone, type A (Silastic®). Implants were incubated in sterile saline, sterilized with ethanol and rinsed with saline prior to implantation.

For rats in the hormone-primed groups, silastic implants were inserted subcutaneously between the scapula under isoflurane anaesthesia according to the following schedule. One week post-ovariectomy, Experimental Day 1, each rat in the three hormone replacement groups was implanted with one 2 mm 17*B* estradiol-filled silastic capsule. Two days later, on Experimental Day 3, 3 progesterone-filled capsules were implanted at the same site. On Experimental Day 14, the progesterone implants were removed.

### Behavioral Testing

Rats in the HP Not Maternal and HP Maternal groups were exposed to pups one day following progesterone removal as described in [17]. Hormonally primed virgin females were presented with three 2–5 day old pups in their home cage at 8 am every morning by placing one pup in each of the quadrants away from the female. Positions of the female and pups were noted every 15 minutes for the first hour of every day and then once an hour for every subsequent hour until 4 pm, when pups were removed from the cage and observation ended. When a female was found to be in proximity of the pups (in the same quadrant) then the pups were scattered and the female observed for maternal behavior. An animal was deemed maternal when she would retrieve the pups, group them, and crouch over them. Once maternal, animals were allowed three hours of experience with the pups and then perfused; forming the HP Maternal group. Hormonally primed rats matched for amount of pup exposure but that had not yet shown any maternal behavior formed the HP Not Maternal group and were perfused at the same time as rats in the HP maternal group.

In order to control for the length of time elapsed since removal of the progesterone implant, one rat from the HP group was also perfused at this time.

### Perfusion and Immunocytochemistry

All rats received an overdose of sodium pentobarbital and were transcardially perfused (descending aorta clamped) using 150 ml of 0.9% saline and 150 ml of 4% paraformaldehyde in 0.1 M phosphate buffer (Sigma, Canada). Brains were removed and postfixed in 30% sucrose in 4% paraformaldehyde made in 0.1 M PB for 48 hr and stored at −80 degrees Celsius until sectioning. Three sets of 40-micron sections were collected through the areas of interest (corresponding to Plate 17 to Plate 20 of Paxinos and Watson's (1986) Atlas of the Rat Brain) using a cryostat. Sections were stored at −20 degrees Celsius in Watson's Cryoprotectant solution until processing for immunocytochemistry.

#### S-100beta, GS and Vimentin Immunocytochemistry

At the time of processing, sections were rinsed in Tris-buffered saline (TBS, Sigma) 3 times for 10 min each, incubated in primary antibody solution [1∶1000, anti-S-100beta (Sigma Canada); 1∶500, anti-glutamine synthetase (Chemicon); 1∶1000, anti-vimentin (Sigma, Canada, Clone v9)] 0.3% Triton X-100 TBS (Sigma), 3% Normal Goat Serum (NHS, Vector Laboratories, USA) and stored overnight at 4 degrees Celsius. Sections were then rinsed 3 times for 5 minutes in TBS and incubated for one hour at room temperature in a secondary antibody solution (1∶200 Anti-rabbit IgG (Vector Laboratories), 1.5% NHS and TBS) or for S100beta images 1∶500 in Alexa Fluor 488 (Invitrogen) in 3% PBST. For diamino-benzidine-2,3 (DAB) staining the sections were then rinsed again in TBS and processed in an avidin-biotin complex solution (ABC Elite kit, Vector Laboratories) containing 0.2% Solution A and 0.2% Solution B diluted in 0.1 M TBS for 30 min at room temperature. Finally, sections were washed again in TBS 3 times for 5 min and immersed in DAB using a DAB-peroxidase kit (Vector Laboratories) omitting the nickel chloride step to reveal a deep brown stain.

### Microscopic analysis

Sections were viewed using a Leica DMR-HC microscope mounted with a Hitachi 3CCD camera (Model #HV-C20) and images of Cg2 and MPOA captured on a G4 Macintosh computer using Scion Image Software 1.66. Images of the entire Cg2 and MPOA (as identified in Paxinos and Watson's (1986) atlas of the rat brain- plate numbers 17–20 (Cg2) 18–20 (MPOA) were taken for at least three sections per animal (Average 4/rat), at a 40× magnification. The lateral extremity of Cg2 was defined by the most dorsal tip of the corpus callosum, directly above the area known as the glial sling, the central sulcus defined the medial line extremity, and dorsally, by a line half way between the corpus callosum and the top of the brain. For each rat, the number of cells positive for S-100beta, GS and Vimentin that displayed a cell body and processes corresponding to the morphology of protoplasmic or radial astrocytes, were counted in all images and averaged across sections. All image analysis and cell counting were carried out by an individual blind to group membership.

### Quantification of Glutamate and Glutamine levels

Rats were decapitated and brains removed, fast frozen and stored at −80°C. Punches of frozen tissue were collected from both hemispheres of the Cg2 and the parietal cortex (ParCx). Punches were suspended in 100 µl of phosphate buffer (0.1 M) and frozen at −80°C for 20 min. The samples were then thawed at 4°C, vortex for 45 sec and centrifuged at 10000 r.p.m. for 10 min. The supernatant was removed and assayed for glutamate and glutamine using high-performance liquid chromatography (HPLC) with fluorescence detection. Samples were loaded by an autosampler (AS3000, Lab Alliance, Fisher Scientific, Montreal, Quebec, Canada) that performed a precolumn derivatization with o-phthalaldehyde. Amino acids were separated with a C-18 reverse-phase column (5 µm, 15 cm, 4 mm ID, Higgins Analytical). Glutamate and glutamine was detected using Ultrafluor (SN4000, Lab Alliance, Fisher Scientific, Montreal, Quebec, Canada) with an excitation wavelength of 340 nm and emission wavelength of 450 nm. The concentrations were estimated from peak height by comparison with injection of known amounts of pure standards (Sigma).

Pellets were suspended in 0.1-M NaCl and analyzed for protein content using spectrophotometry (Pharmacia Biotech Novaspec II, wavelength 595 nm) to quantify protein binding with Coomassie Brilliant Blue G (Sigma). Amount of proteins present in pellets were derived from a standard curve obtained with Bovine serum albumin (Sigma). The final levels of Glutamate and Glutamine are expressed as picograms per milligram of protein. Protein levels from both hemispheres were assessed separately in order to control for possible lateralization effects, however, data was collapsed across hemispheres if no hemispheric differences were observed.

### Western Blotting

Rats were decapitated and brains removed, fast frozen and stored at −80°C. Punches of frozen tissue were collected from both hemispheres of the hippocampus, Cg2, and ParCx. 120 µl of Lysis buffer was added to each sample and protein was homogenized using a sonicator. Samples were then centrifuged and the supernatant collected and stored at −20°C. Protein analysis was conducted using the BCA-200 Protein Assay Kit (Pierce) according to manufacturers instructions and 40 µg of protein was loaded into wells of 12% SDS-PAGE (NuPAGE- Invitrogen) gels along with the sample buffer, reducing agent (Invitrogen) and water (if needed) to a total volume of 14 µl/ well. All buffers were obtained from Invitrogen and used with the PowerEase TM 500 from Invitrogen. Gels were run at 110volts for 15 minutes followed by 150volts for one hour and then transferred to nitrocellulose membranes (Invitrogen) at 100volts for 1 hour. Ponceau S (Sigma Canada) was used to verify successful transfer of proteins. Membranes were then washed in a blocking and incubation buffer and incubated in a primary antibody solution at 4°C overnight GLT1 (1∶2,000 Chemicon), GLAST (1∶1,000 Chemicon) On the following day, membranes were washed and incubated in appropriate hrp-conjugated secondary antibody for 2 hours, washed and processed using Chemiluminescence Reagent Plus (Perkin Elmer Life Sciences) detection kit, following the manufacturers directions. Images were captured using Kodak ID Image Station and Software.

### Data analysis

A one-way between groups analysis of variance, ANOVA, was used to analyze the differences across treatment groups for each brain area and each protein under investigation. The level of significance was set at p£ .05 and, if significant, post-hoc pairwise analyses were conducted using Fisher's LSD.

## Results

### Glial Markers

**Figure 1:**
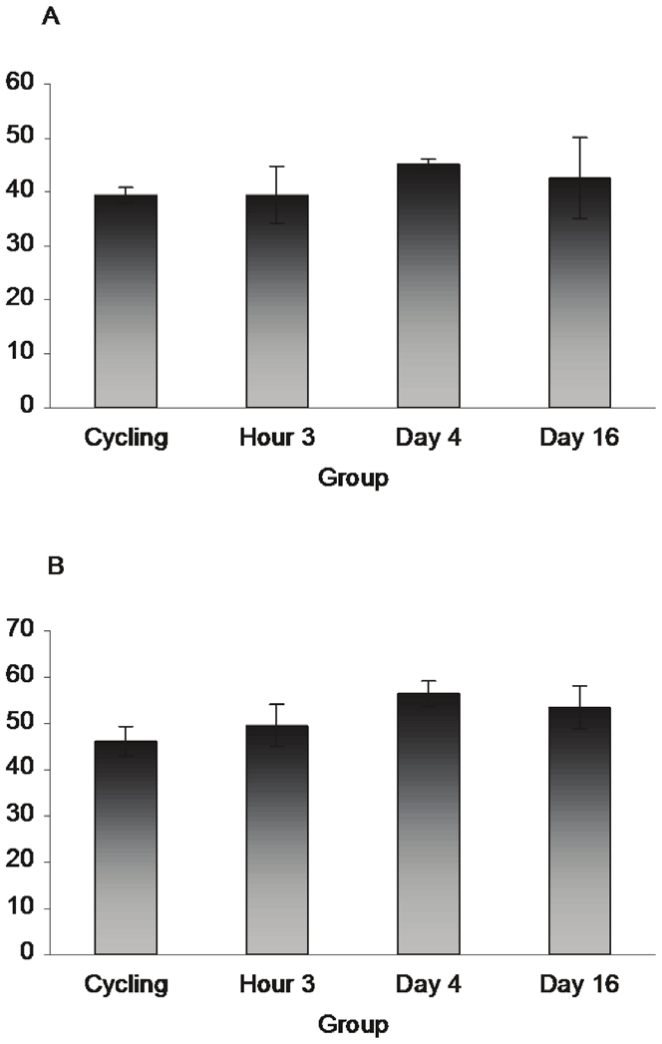
S100beta does not vary with reproductive state. Panel A shows the mean number of S100beta immunoreactive cells in Cg2 and MPOA (B) in cycling and 3 hours, 4 and 16 day postpartum animals. No significant differences across groups were observed in S100beta levels. However, unlike bFGF and GFAP, S100beta did label astrocytes within the Cg2 of cycling animals. (Cg2: cycling n = 4, H3 n = 4, D4 n = 5, D16 n = 6; MPOA: cycling n = 5, H3 n = 4, D4 n = 4, D16 n = 5)

**Figure 2:**
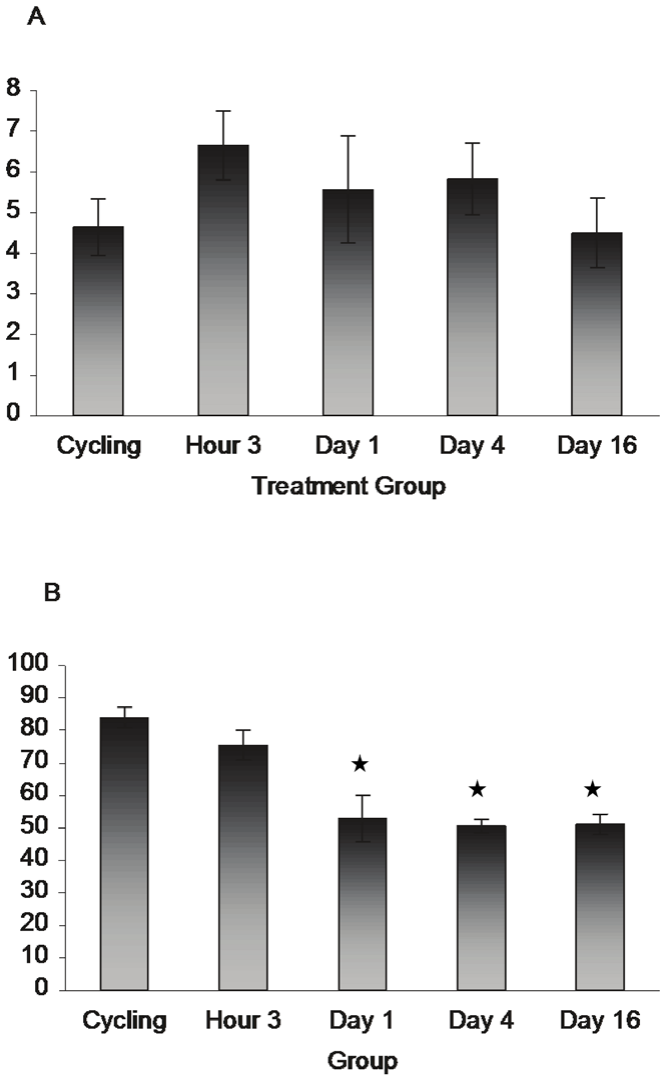
Representative photomicrographs of vimentin-ir in the MPOA. Panel A shows vimentin-ir of the MPOA of Cycling animals, and Day 16 (MPOA- panel B, Cg2 Panel C). Note the decrease in vimentin-ir within the MPOA during lactation and the relative absence of vimentin-ir cells within Cg2. Panels D and E show darkfield images of S100beta-ir within Cg2 of Cycling (D) and D16 (E) and in the MPOA of an H3 postpartum rat (F).

S100beta- *As* *Figures 1A and B* *show there was no effect of reproductive state on the number of S100beta-ir cells in either Cg2 (F_3,15_ = 2.166, p>.05) or in the MPOA (F_3,15_ = 1.307, p>.05), see also images in* *Figure 2* *.*

**Figure 3:**
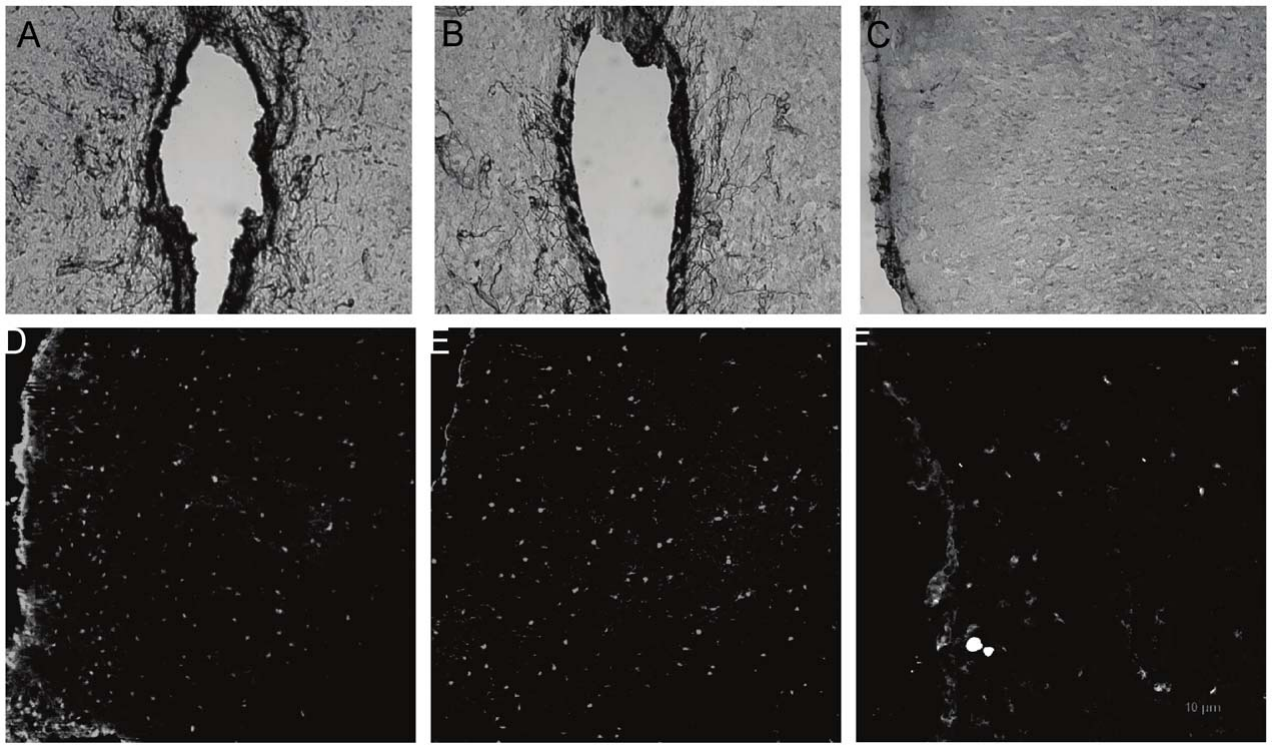
Vimentin-ir cells within the Cg2 and MPOA of cycling and postpartum animals. No significant changes were observed within Cg2 (A), however, vimentin-ir remained decreased in the MPOA (B) after 24 hours postpartum, suggesting a decrease in immature radial glia in this area during the postpartum period. Asterisks denote a significant difference (p<.05) from all other groups. (Cg2: cycling n = 3, all other groups, n = 4 MPOA: n = 4 for all groups)

Vimentin- No significant differences were observed among treatment groups in levels of vimentin-ir within Cg2 (F _4,15_ = .831, p>.05). Significant effects were found overall within the MPOA (F _4,15_ = 13.162, p<.0001), post-hoc comparisons revealed that postpartum rats in the Day 1, 4, and 16 groups showed lower levels of vimentin than the cycling and the H3 groups (see Figures 3A, 3B and 2).

**Figure 4:**
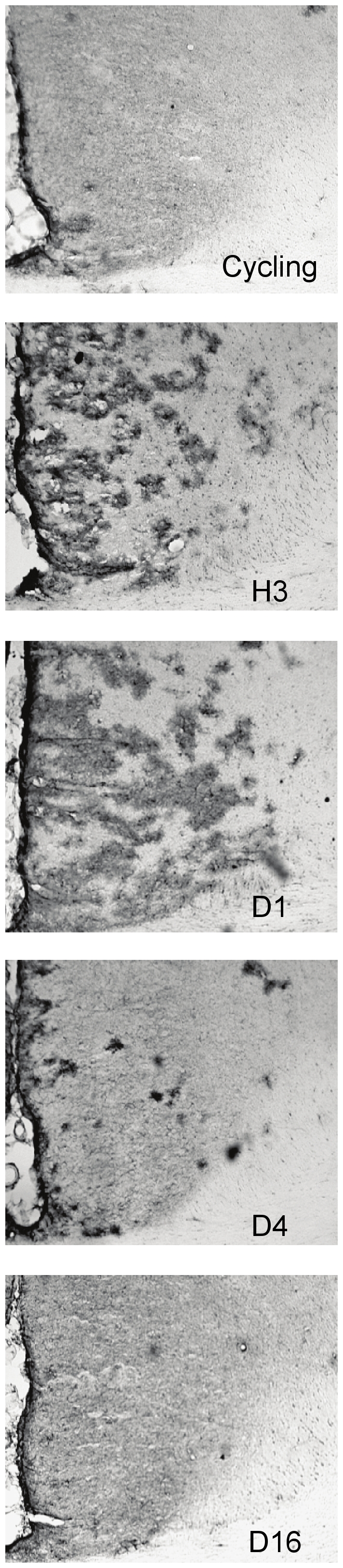
Representative sample of photomicrographs of GS-ir staining within Cg2. Note the upregulation of GS-ir in Day 1 postpartum animals.

**Figure 5:**
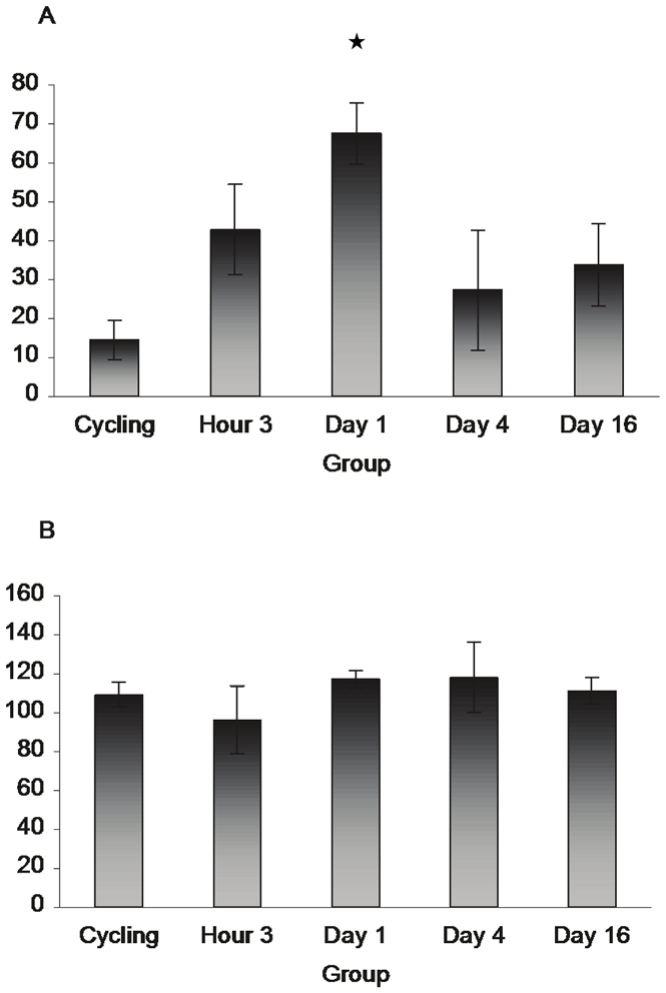
Reproductive state changes Glutamine Synthetase-ir within Cg2. Panel A shows results from the Cg2 and panel B the MPOA. Asterisks denote a significant difference (p<.05) from all other groups. (n = 4 for all groups/area).

**Figure 6:**
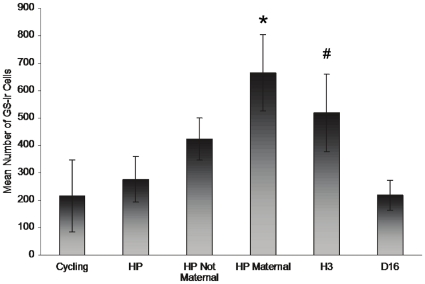
Hormones and maternal behavior together stimulate GS-ir within Cg2. The HP Maternal group was significantly higher from both the cycling and HP groups. The H3 group showed significantly elevated levels of GS-ir from cycling animals as well. (Cycling n = 5, HP n = 4, HP Not Maternal n = 6, HP Maternal n = 4, D16 n = 5, H3 n = 5)

Glutamine synthetase- Figure 4 shows examples of staining for GS-ir from all groups. Reproductive state had a significant effect on GS-ir within Cg2 (F_4,15_ = 3.471, p = .03), see Figure 5A. GS- ir was significantly higher in the D1 group than in the cycling, D4 & D16 groups (p<.05) and levels in the H3 group did not differ significantly from any other groups although there was a non significant trend (p<0.07) for this group to have higher levels of GS-ir than cycling rats. As can be seen in Figure 5B, there was no significant change in GS-ir in the MPOA at any of the time points examined. As shown in Figure 6, mimicking the hormonal profile of late pregnancy in ovariectomised rats was not sufficient to induce GS, but hormone treatment in conjunction with 3 hours of maternal experience was sufficient to upregulate GS in Cg2 to the levels seen in postpartum animals (F(5,23) = 2.63, p = .05).

### Glutamate Transporters

**Figure 7:**
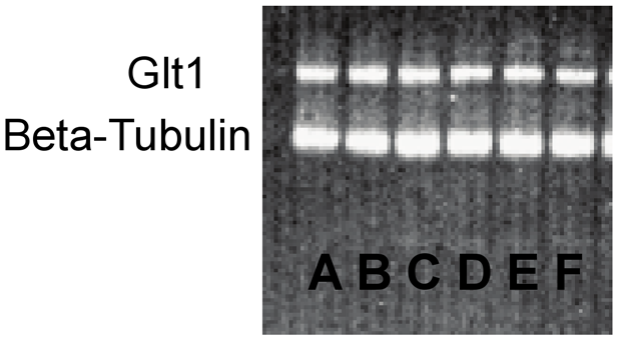
A representative blot image of GLT-1 and beta-tubulin from Cg2. Cycling (A), Mid-Pregnant (B), Late Pregnant (C), Hour 3 (D), Day 1 (E), and Day 16 postpartum (F). No significant differences were observed between groups.

No effect of reproductive state on either glt-1 or glast protein expression was observed in western blots of the cingulate or parietal cortices (see Figure 7 for a representative western blot image).

### Quantification of Glutamate and Glutamine

**Figure 8:**
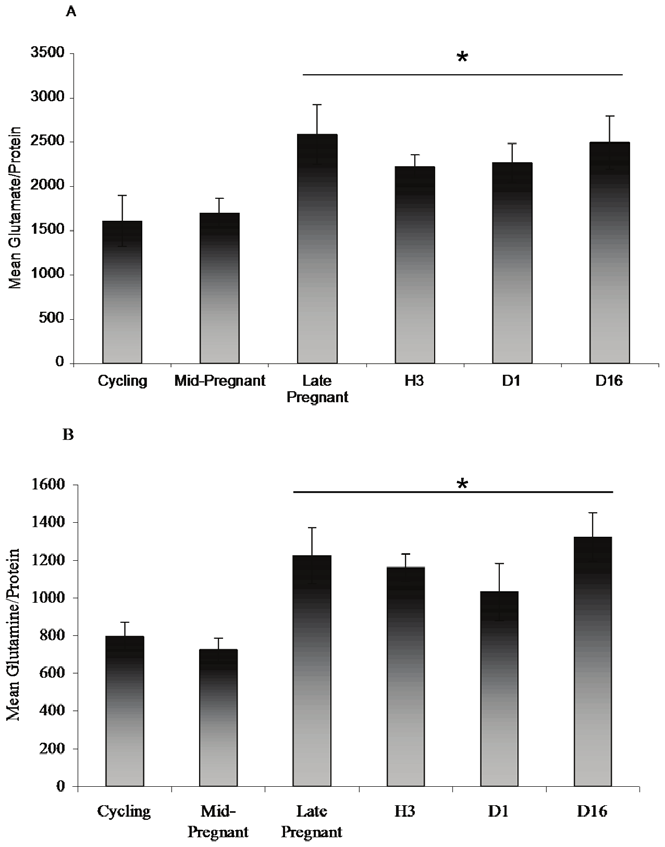
Levels of glutamate and glutamine protein within the Cg2 change with reproductive state. Glutamate (A) or glutamine (B) levels were divided by total protein in picograms per microliter. Asterisks denote a significant increase from cycling and mid-pregnant groups. (Cycling, D1, D16 n = 5; Mid-pregnant, Late Pregnant and Hour 3, n = 6)

Levels of glutamate and glutamine within the Cg2 and parietal cortex of cycling, pregnant and lactating rats were assessed by HPLC. Results showed a significant overall effect of reproductive state on both glutamate and glutamine protein levels within Cg2 (glutamate:F(5.27) = 2.668, p<.05; glutamine:F(5,27) = 4.683, p<.01) (see Figure 8A & B, respectively). Late pregnant and postpartum groups had significantly higher glutamate and glutamine levels than the cycling and mid-pregnant groups. No significant effect of reproductive state on glutamate or glutamine protein levels were observed in the parietal cortex (data not shown) suggesting that the changes observed in Cg2 did not reflect a generalized upregulation of glutamate/glutamine throughout the cortex.

## Discussion

In previous studies we have consistently shown an upregulation in the number of GFAP-ir cells in area Cg2 of lactating females [11], [12], [13], [17], [23]. In the current paper, we elaborated on these findings by comparing immunoreactivity for three less common markers of astrocytes; S100beta, GS and vimentin, within Cg2 and MPOA amongst groups of cycling and lactating females. The number of mature astrocytes observed, as reflected in the number of cells showing S100beta-ir, did not change across reproductive states in either of the areas examined. These data suggest that the absence of GFAP-ir cells that we have observed in cycling rats in previous studies is indicative of a change in the functional state of the astrocytes in this area rather than in astroglial cell number. There were changes in patterns of expression of both vimentin and GS across reproductive states, however, and these differed between the areas examined.

Vimentin is an intermediate filament protein that is highly expressed in radial glia and immature astrocytes. We found that very few cells in Cg2 expressed vimentin and this number did not fluctuate with reproductive state. Given that vimentin is expressed most robustly during development it may well be that levels of vimentin-ir are indeed unaffected by reproductive state. Alternatively, it is possible that we observed no changes in the expression of this protein simply because the adult brain does not signify an optimal time point for observation of this protein- at least in the cortex. During adulthood, vimentin is expressed transiently and typically only prior to expression of other intermediate filament proteins, and typically in neurogenic regions such as the sub-ventricular and sub-granular zones [24]. The time points that we examined in the current study were ones at which we knew GFAP would be elevated and thus potentially too late to observe an upregulation of vimentin, if any occurs at all within this region. Interestingly, we did observe changes in vimentin expression in the MPOA. Rats in the cycling and H3 postpartum groups showed higher numbers of vimentin-ir cells than the other groups. The factors that might induce such a suppression are not clear. Nor are the effects on neural functioning. However, increased vimentin expression is typically observed in astroglial stem cells and is associated with periods of increased plasticity and neurogenesis [21].

GS-ir did not change across groups within the MPOA but there were significant changes associated with reproductive state in Cg2. Peak levels of immunoreactivity were expressed on D1 postpartum, with a return to those levels seen on the metestrus day of the cycle by D4 of lactation. GS, like S100beta, is used as a marker of mature astrocytes. The fact that, in Cg2, we observed different patterns of expression of these two markers across reproductive states may reflect their very different functions [20], [22], [25]. S100beta is a calcium binding growth factor important in the stimulation of neurite extension length [22], [25]. Over-expression of this protein has been associated with brain damage and conditions such as Alzheimer's disease [22], [26], [27]. GS, on the other hand, is important for the breakdown of glutamate to glutamine and plays a role in regulating levels of extracellular glutamate. GS activity can be modulated by glutamate, NMDA receptor activation and nitric oxide [28].

We also found that both pregnancy hormones and maternal experience were necessary to induce upregulation of GS. Interestingly, those animals that were hormonally primed and exposed to pups but that were not yet maternal (HP Not Maternal) did not differ significantly from either the HP or HP maternal groups, perhaps because this group was transitioning towards the levels of GS upregulation seen in the maternally experienced group. These data are consistent with our earlier findings that both hormonal state and maternal experience are critical in inducing changes in astrocytic proteins in Cg2.

Together these data could indicate that the hormonal changes of late pregnancy combined with the olfactory, tactile and suckling stimulation associated with maternal experience induce an increase in glutamate within Cg2 that initiates both upregulation of GS and bFGF. Indeed, results from our HPLC analysis showed that both glutamate and its metabolite glutamine are upregulated at the end of pregnancy and remain upregulated throughout the lactating period. In addition, it has been well-documented that increased glutamate release typically induces changes in glutamate metabolism in surrounding astrocytes, which would be congruent with the changes we have observed in bFGF, GFAP and GS expression within Cg2 of the postpartum rat. Indeed, it has been suggested that bFGF expression is stimulated by glutamate (Flores and Stewart, 2000) and, consistent with this hypothesis, bFGF-ir in Cg2, like glutamate concentrations, remains high throughout lactation. By contrast, the current data show only a transient increase in expression of GS. Kruchkova et al., (2001) found that bFGF acting via a c-jun pathway actually downregulated GS production. Thus, it is possible that hormonal state and pup stimulation combined increase glutamate release into Cg2 that, in turn, stimulates both increased bFGF and GS expression. This continued upregulation of bFGF is able to then suppress GS production. One issue yet to be investigated is the pathways through which increases in glutamate concentration are themselves induced. We have previously shown that there is no postpartum induction of immediate early genes in Cg2, suggesting no increase in neuronal activity within this area. However, we [29], [30] did observe increased immediate early gene activity in the early postpartum period within several regions that send important projections to Cg2 including the parietal cortex and MPOA [31], [32], [33]. The increased activity in these areas may lead to the increased glutamate concentrations in Cg2 that we have observed in late pregnancy and during the postpartum period.

The question as to the functional (behavioral) outcome of post-partum changes in glia and neurons within Cg2 remains, however. Interestingly, several early studies in both mice and rats have pointed to the medial frontal cortical area (including the region of the cingulate investigated in our studies) as necessary for the organization of maternal behavior. Mice and rats with lesions of this area were not impaired on the “motivation” to show maternal care (avidly retrieving their pups) but rather showed disorganized and dysregulated behavior (dropping and re-retrieving the pups and building disorganized nests) that ultimately led to pup neglect and death. It is unlikely that the Cg2 is specifically critical for the expression of maternal behavior, but rather, we propose that the cingulate cortex's general role of organizing and sequencing behavioral is critical for successful maternal behavior. Needless to say this idea is largely theoretical, and it remains to be seen whether blocking the neuronal/glial changes that we have observed in Cg2 would affect maternal behavior as do lesions to this region.

In sum, we have consistently observed increases in astrocytes that co-express GFAP and bFGF in Cg2 during the postpartum period [11], [12], [23]. The current data suggest that this upregulation is confined only to a subset of astrocytic markers suggesting that it is the state rather than the number of astrocytes that is modulated in this area by reproductive state. Moreover, we found that the peripartum and lactational period is associated with upregulation of astrocytic proteins involved in glutamate metabolism and in glutamate itself as well as its metabolite, glutamine. Together with our previous results, the current data support the notion of specific changes in Cg2 and provide a potential mechanism through which such changes might occur. These results also emphasize the usefulness of using multiple glial markers in understanding astrocytic modifications across reproductive states. Whether the net effect of the changes that we have observed in Cg2 is to maintain neural activation at levels seen in non-lactating rats or whether they are part of a reorganization of pathways in this area remains to be determined.
